# A hundred spotlights on microbiology: how microorganisms shape our lives

**DOI:** 10.15698/mic2022.04.773

**Published:** 2022-04-04

**Authors:** Didac Carmona-Gutierrez, Katharina Kainz, Andreas Zimmermann, Sebastian J. Hofer, Maria A. Bauer, Christoph Ruckenstuhl, Guido Kroemer, Frank Madeo

**Affiliations:** 1 Institute of Molecular Biosciences, NAWI Graz, University of Graz, Graz, Austria.; 2 Centre de Recherche des Cordeliers, Equipe labellisée par la Ligue contre le cancer, Université de Paris Cité, Sorbonne Université, Inserm U1138, Institut Universitaire de France, Paris, France.; 3 Metabolomics and Cell Biology Platforms, Institut Gustave Roussy, Villejuif, France.; 4 Institut du Cancer Paris CARPEM, Department of Biology, Hôpital Européen Georges Pompidou, AP-HP, Paris, France.; 5 BioHealth Graz, Graz, Austria.; 6 BioTechMed Graz, Graz 8010, Austria.

**Keywords:** infectious diseases, model organism, yeast, biotechnology, microbiome, microbiota, symbiosis, open access

## Abstract

Viral, bacterial, fungal and protozoal biology is of cardinal importance for the evolutionary history of life, ecology, biotechnology and infectious diseases. Various microbiological model systems have fundamentally contributed to the understanding of molecular and cellular processes, including the cell cycle, cell death, mitochondrial biogenesis, vesicular fusion and autophagy, among many others. Microbial interactions within the environment have profound effects on many fields of biology, from ecological diversity to the highly complex and multifaceted impact of the microbiome on human health. Also, biotechnological innovation and corresponding industrial operations strongly depend on microbial engineering. With this wide range of impact in mind, the peer-reviewed and open access journal *Microbial Cell* was founded in 2014 and celebrates its 100^th^ issue this month. Here, we briefly summarize how the vast diversity of microbiological subjects influences our personal and societal lives and shortly review the milestones achieved by *Microbial Cell* during the last years.

## THE MANY IMPLICATIONS AND CHALLENGES OF MICROBIAL RESEARCH

**Figure 1:**
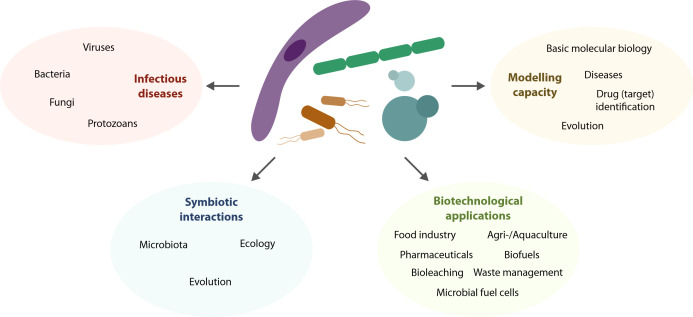
FIGURE 1: The multilayered impact of microorganisms on our lives.

The history of life on Earth is mainly microbial. The emergence of the first microorganisms 3-4 billion years ago [1] was the initial step for the establishment of terrestrial life. Microorganisms critically contributed to our planet's transformation, with the rise of photosynthetic bacteria allowing for oxygen to build up in the atmosphere [2]. Nowadays, microorganisms continue to affect the planet's biosphere and are an integral and inextricable part of our lives at different levels. The exploration of the microbial world is not only key to understanding ourselves but can provide answers to many medical, technological and scientific questions we face as humankind. Here, we briefly summarize the main areas, on which microorganisms impact today and will in the future, in particular (i) infectious diseases, (ii) symbiotic interactions, (iii) biotechnological applications and (iv) biological models (**Figure 1**).

## INFECTIOUS DISEASES

Infectious diseases continue to threaten our lives and societies, as revealed by the current COVID-19 pandemic. This applies in particular to persisting, emerging and re-emerging infectious diseases. In that respect, it will be important to monitor the human-animal interface, and recognize that many emerging diseases are zoonotic, i.e. they spread between animals and humans and have an animal reservoir [3]. This threat emanates from all microbial phyla. Apart from minatory viral infections [4], bacterial and fungal infections continue to cause millions of deaths worldwide [5, 6]. An alarming rise in antibiotic and antifungal resistance exacerbates this menace [5, 7], representing one of the most acute challenges in medical microbiology. Finally, protozoan infections remain a public health threat associated with significant morbidity and continue to have a substantial socioeconomic impact [8, 9]. The limited advancements in vaccination strategies and the increasing parasite resistance against existing drugs further exacerbate the problem [10]. Also, coinfections of different pathogens belonging to one or different species can occur, resulting in additional challenges [11–14]. Thus, more work is needed to explore the molecular and pathological interaction between co-infecting microorganisms. This applies to both the direct interplay with each other and the indirect interaction through the host, for instance via the immune system. Altogether, infectious diseases continue to be devastating despite the many medical improvements accomplished in the last decades. Of note, many microbial pathogens also pose a threat to agriculture and livestock farming [15, 16], which further aggravates the socioeconomic burden of infectious diseases.

## SYMBIOTIC INTERACTIONS

The participation of microorganisms in symbiotic interactions determines a vast range of biological aspects across species. In particular, the human gut microbiota – the collection of bacteria, archaea and eukarya colonizing the gastrointestinal tract [17] - has attracted much attention in the past decade. The gut microbiota has a deep impact on many instances of human biology, ranging from development, physiology and immune homeostasis to health, nutrition and even behavior [18–21]. In addition, the importance of the human virome is increasingly being recognized. The human virome is composed of bacteriophages that infect bacteria, viruses that infect other cellular microorganisms (archaea, eukarya), viruses that infect human cells and some transitory viruses originating in food [22]. Their interactions with the human host are only beginning to be understood, but clearly hint towards a decisive role in health (e.g. via interplay with the host immune system) and multiple diseases (e.g. diabetes, hypertension and cancer) [22]. From a broader perspective, microbial symbiosis affects and co-defines a vast array of ecological aspects, ranging from plant growth [23] to defensive capacities [24]. For instance, complete ecosystems like hydrothermal vents and coral reefs exemplify the ecological success of microbial-multicellular symbioses. Finally, symbiosis can uniquely drive evolutionary innovation; this is maybe best embodied in the endosymbiotic origins of mitochondria and chloroplasts in eukaryotic cells [25, 26], where the symbiont was cellularly and genomically integrated into the host.

## BIOTECHNOLOGY

From a historical or even pre-historical perspective, already in ancient times, microorganisms were used as tools to produce, ferment or process a diversity of important food items including vinegar, bread, beer, fish, cheese and wine [27]. In other words, microorganisms were actively used as biotechnological agents long before the scientific basis underlying these processes was even known. Nowadays, microorganisms represent an essential backbone of many biotechnological applications thanks to their rapid growth for quick production, technical versatility for production design and wide applicability to a number of industrial sectors. Indeed, fundamental and applied microbiology are essential components of modern biotechnology with an ever-increasing economic impact. For instance, the food industry heavily relies on microorganisms for applications that range from fermented food items and alcoholic beverages to food grade components and bio-based ingredients in general. Thus, bacteria are used for the production of thickening or gelling agents, flavor compounds and enhancers, acidulants, vitamins and colorants [28]. Moreover, microorganisms have a deep impact on biotechnological approaches in agriculture and aquaculture with developments ahead that may use host-microbe interactions and the host microbiome for sustainable production [29]. Microorganisms also play a significant role in environmental biotechnology, including municipal and industrial water waste management [30] as well as treatment of solid hazardous waste [31]. Other applications involve the treatment of oil spillage [32], radioactive contamination [33], electronic waste processing [34], bioleaching (the extraction of metals from their ores through the action of microorganisms) [35] or even space biomining [36]. Yet another economically relevant use is energy production. Biofuels are produced by engineered microorganisms that utilize renewable carbon sources. Although they have shown great potential in replacing fossil fuels (especially ethanol and biodiesel), there are still some limitations, including applicability in conventional engines and high costs [37]. So-called microbial fuel cells (MFCs), which use bacteria to oxidize organic and inorganic matter in order to generate current, may represent an appealing electrogenic approach in the future [38]. Another example of microbial biotechnology is pharmaceutical production, which includes heterologous expression of human proteins, microbial enzymes or drug compounds for medical and research purposes [39]. Some of the aforementioned examples are established processes while others are still in development, revealing the huge potential and economic impact of microorganisms in technological approaches [40].

## MODELLING BASIC PRINCIPLES OF BIOLOGY

The short generation time, facile cultivation and ease of genetic manipulation have established a number of microorganisms as widely used model organisms. *Escherichia coli* has been instrumental in the discovery and understanding of basic molecular biology processes, including DNA replication, DNA-to-RNA transcription and the genetic code allowing for RNA-to-protein translation. To date, a total of twelve Nobel Prizes were awarded for work that used *E. coli* as a research organism or tool, and its potential to assist in further advancements remains high [41]. A number of other prokaryotes (including archaea [42]) are actively used as model organisms. For example, *Bacillus subtilis* is applied to study biofilms, bacterial asymmetry or morphogenesis [43], cyanobacteria like *Synechocystis* sp. PCC 6803 to model photosynthesis [44], or *Caulobacter crescentus* for the study of cellular differentiation, motility or mechanosensing [45]. Green algae, including unicellular *Chlamydomonas reinhardtii* and multicellular *Volvox carteri* (Volvox), also serve as model organisms. *C. reinhardtii* bears both animal-like organelles (cilia) and plant-like organelles (chloroplasts), allowing research into the function of flagella and photosynthesis [46]. Volvox is mainly used to investigate developmental mechanisms and the evolutionary origins of multicellularity [47]. Other protists include the giant heterotrichous ciliate *Stentor coeruleus as a model for cellular regeneration and wound healing* [48] and the ciliate *Oxytricha*, which is employed in the areas of genome biology, post-zygotic development and epigenetic inheritance [49]. The eukaryotic nature of these cells allows for the study of essential and medically relevant molecular processes. Similarly, yeast cells display all advantages of unicellular model organisms paired with a high degree of conservation that has made yeast a fundamental partner in elucidating many aspects of human physiology and pathology [50]. Work performed in yeast has been awarded five Nobel Prizes in the past two decades. The budding yeast *Saccharomyces cerevisiae* is used to study a multitude of human diseases (e.g. neurodegeneration, cancer) [51, 52], characterize basic physiological processes (e.g. cell death, aging, autophagy, mitochondrial import, vesicle fusion, cell cycle) [53–64] and identify novel medical drugs (e.g. antiaging, anticancer, antiparasitic, antifungal) [56, 65–70], among many other applications [71]. Another example is the fission yeast *Schizosaccharomyces pombe*, which is used to analyze, for example, cell cycle processes or DNA checkpoints [72, 73].

## A PUBLICATION PLATFORM FOR MICROBIOLOGICAL RESEARCH

Given the many layers of how microorganisms wield huge influence on our lives, the open-access journal *Microbial Cell* was founded with the idea to generate an online *agora* for all types of research in the microbiological field. The current issue (Volume 9, Issue 4) marks a milestone in *Microbial Cell*'s history: it represents the 100^th^ issue since the journal was launched in January 2014. This occasion is a timely moment to take stock and reflect on how *Microbial Cell* has developed and contributed to the research fields of unicellular and multicellular microorganisms over the past years.

The mere fact that a journal run by active academic scientists and through an independent publisher (Shared Science Publishers) has established itself in the highly competitive business of peer-reviewed scientific publishing is *per se* a great achievement. That this has occurred in a radically open-access fashion and in such an important and trending field like microbiology adds even more value to this accomplishment. We take this opportunity to thank all authors who have published their articles in *Microbial Cell* for their trust put in the journal to run the evaluation and dissemination processes of their work. At the same time, we congratulate the authors for the high quality of their papers: the around 400 articles that have been published since the journal was launched have now cumulatively been cited more than 4000 times (Web of Science, Clarivate Analytics).

We acknowledge all members of the Editorial Board for their long-standing commitment and reliability. Indeed, our Editorial Board has the arduous task to evaluate submissions in ten different thematic subareas: aging, cell death, cell physiology and cell signaling, genome stability and structure, infection biology, microbiome, mitochondria, parasitology, stress response, and structural & systems biology. During the manuscript evaluation process, these editors strongly rely on the expertise and rigor of peer reviewers, who invest a great amount of their time in improving the submitted work. We are very grateful to all our referees throughout the world for their invaluable input. The combined effort of all partners of the ecosystem, authors, editors and reviewers, has consistently improved the journal throughout these years to place it at the apex of microbiology.

## A BRIEF HISTORY OF MICROBIAL CELL

*Microbial Cell* emerged as an academic effort from a group of active scientists to apprehend the thematic heterogeneity of microbial research. Accordingly, the inaugural Editorial from January 2014 outlined that *Microbial Cell* would have the mission to facilitate “the characterization of unicellular organisms (or multicellular microorganisms) in their response to internal and external stimuli and/or in the context of human health and disease” [74]. This definition mirrored well the initial and still persisting idea of a publication platform that acknowledges microbiological interdisciplinarity.

Following these objectives, the first 100 issues of *Microbial Cell* have accompanied and contributed to several developments in microbiological research during the last eight years. For instance, in a much regarded *Microbial Cell* paper of 2015, Alexander Varshavsky and colleagues described formylated N-terminal methionine as a novel bacterial degradation signal used in a new branch of the bacterial N-end rule pathway [75]. Varshavsky's lab was instrumental in the discovery of the ubiquitin system of intracellular protein degradation and was the first to describe the connection between the N-terminal residue of a protein and its half-life [76, 77]. As another example, in 2016 *Microbial Cell* published a highly cited review, in which Daniel J. Klionsky and colleagues provided a brief but comprehensive summary on the roles, regulatory instances and molecular mechanisms of autophagy [78]. 2016 was also the year, in which Yoshinori Ohsumi was awarded the Nobel Prize in Physiology or Medicine for his discovery of the mechanisms orchestrating this intracellular degradation pathway, which were uncovered in yeast cells [79–81]. In 2018 *Microbial Cell* published a review by Francisco J.M. Mójica and colleagues that provided an overview of the CRISPR-Cas mechanism as a prokaryotic immune system and discussed a number of evolutionary implications [82]. Mójica's achievements, particularly his groundbreaking work characterizing CRISPR (Clustered Regularly Interspaced Short Palindromic Repeats) loci [83, 84], laid the ground for the development of CRISPR-Cas as the most important tool for genomic editing that exists to date. In 2020, Emmanuelle Charpentier and Jennifer A. Doudna received the Nobel Prize in Chemistry for the development of this method [85]. One final example for how *Microbial Cell* has contributed to research deals with severe acute respiratory syndrome coronavirus 2 (SARS-CoV-2), which causes the highly infectious disease COVID-19. The multilayered consequences of COVID-19 at the individual, social, political and economic levels are unprecedented in our globalized world. *Microbial Cell* has been publishing diverse papers on SARS-CoV-2, ranging from mechanistic viewpoints to methodological approaches for viral detection and molecular modelling.

The last 100 issues have seen *Microbial Cell* grow as a journal and establish itself in the microbiology publishing sphere. The journal's CiteScore (Scopus) is currently 7.1 (provisional score for 2021), which ranks the journal at top positions in all relevant microbiology-related categories. *Microbial Cell* has established roots in various research communities as highlighted by reviews, research papers, commentaries, as well as by a number of guideline papers that establish unified criteria in a number of fields, for instance in DNA recombination and repair, yeast cell death and antifungal and antibiofilm agents. Furthermore, *Microbial Cell* has initiated the publication of several Special Issues on a diverse range of topics, following a concept, in which incoming papers are published in regular issues and then collected in the Special Issue repositorium. That way, Special Issues can be updated continuously and refreshed by new articles that capture new evolving knowledge. Until now, *Microbial Cell* has launched Special Issues on the following topics [86]: sexually transmitted infections (started in 2016), the human microbiome in health and disease (started in 2019), hygiene in healthcare (started in 2019), microbiology in cultural heritage (started in 2021) and yeast cell death (started in 2022).

In the course of the last eight years, *Microbial Cell* has strengthened its position in the microbiology-publishing landscape by attaining a number of milestones. In 2014, *Microbial Cell* partnered with the World Health Organization's HINARI program to support free access to biomedical research literature. That same year, *Microbial Cell* also became a member of Crossref, an official digital object identifier (DOI) registration agency, providing each article with a persistent interoperable identifier that also enables to precisely link citations across publishers of online academic journals. Also in 2014, *Microbial Cell* was indexed in Sherpa/RoMEO, an aggregator of open access policies of academic journals. In addition, *Microbial Cell* secured a partnership with the Austrian National Library to ensure digital long-term archiving and perpetual access to its complete content. After successful applications to the Chemical Abstracts Service (CAS) and the Directory of Open Access Journals (DOAJ), *Microbial Cell* was accepted in these two renowned repositories in 2015. One year later, *Microbial Cell* was awarded the very selective DOAJ Seal (allocated to only 10% of DOAJ-indexed journals) for best practice in open access publishing. Also in 2016, *Microbial Cell* entered the International Committee of Medical Journal Editors (ICMJE) list to acknowledge that it follows the ICMJE's Recommendations for the Conduct, Reporting, Editing and Publication of Scholarly Work in Medical Journals. That same year, *Microbial Cell* was selected for inclusion in Clarivate Analytics' (formerly Thomson Reuters) Emerging Sources Citation Index (ESCI), thus allowing the journal to be accessed through the Web of Science. This selection subsequently allowed for inclusion into additional Web of Science indexes: Biological Abstracts, BIOSIS Previews, Current Contents Life Sciences and Essential Science Indicators. One of the most important milestones was reached in 2017, when *Microbial Cell* was accepted in Pubmed Central, the archive of biomedical and life sciences journal literature at the U.S. National Institutes of Health's National Library of Medicine (NIH/NLM). After long evaluation periods, *Microbial Cell* was further accepted in two of the most selective indexes, Elsevier's Scopus (2019) and Clarivate Analytics' Science Citation Index Expanded SCIE (2021).

## THE CONCEPT OF MICROBIAL CELL

Over the past few years, *Microbial Cell* has persistently paid high attention to the quality of its published material. Content-related aspects like novelty, methodology, data presentation, appropriate interpretation, etc., are certainly the main denominators of quality in any submission. In addition, the increasing number of scientific misconduct cases requires special attention. That is why *Microbial Cell* implements a very strict and careful evaluation of any submitted material in relation to possible data fabrication, data falsification including inadequate manipulation of images and plagiarism. Thus, each submitted work is tested via CrossCheck, a plagiarism detection service powered by the detection software iThenticate. If concerns are raised, *Microbial Cell* initiates appropriate procedures as detailed by the Committee on Publication Ethics (COPE).

With respect to article accessibility*, Microbial Cell* has always followed an open access approach and used a creative commons (CC) license for copyright purposes. *Microbial Cell* is published under the CC BY license, which is probably the most generous type of CC licenses. The CC BY license authorizes third parties to share and adapt the published work, even for commercial goals, as long as the authors are appropriately credited and changes are indicated. Thereby, the work can be accessed completely free and with no restrictions; the only prerequisite is the connection to the internet, with no other financial, legal or technical limitations. This approach maximizes the visibility of published work and at the same time, ensures that scientific knowledge is universally and freely accessible to every interested individual around the world. This openness reflects the essential values of science and acts as an effective driver of active research, promoting the free exchange of ideas. Incidentally, 2022 marks the 20^th^ anniversary of the Budapest Open Access Initiative, the public statement of principles relating and defining open access to the research literature.

As mentioned, unrestricted article accessibility promotes visibility and increases the impact of a scientific work. The assessment of such an impact involves citations by scholarly journals (because this assessment is peer-connected), which derives in rankings by dedicated indexes. However, we also do acknowledge that alternative outlets, for instance social media, are important indicators of public interest as they increase the diffusion of information at the item (article)-level. Accordingly, *Microbial Cell* actively uses the corresponding channels including Twitter and Facebook. The journal also provides a social impact measure for each article through PlumX Metrics, a comprehensive monitoring tool that calculates altmetrics for scholarly works.

The involvement in, and commitment to, the broad thematic scope of neglected, emerging and trendy microbiology-related topics is a defining characteristic of *Microbial Cell*. As such, the journal has been continuously supporting the efforts of the microbiology research community well beyond its role as a publication platform. For instance, *Microbial Cell* runs a waiver program (DevResearch Program) that allows for the partial or complete exemption of article processing charges for corresponding authors based in low-income or lower-middle-income countries. The journal has also sponsored several prizes at and provided support to international conferences, including the Theodor Escherich Symposium on medical microbiome research, the International Symposium “One mitochondrion, many diseases”, or the International Meeting on Yeast Apoptosis.

Since the genesis of *Microbial Cell*, we have been aware of the plethora of facets that make microorganisms a fundamental part of our lives, including at the historical, medical, diagnostic, evolutionary, ecological, environmental, cultural, biotechnological and modelling levels. In accord with this conviction, we have published 100 issues that reflect the wide-ranging importance of microbial research. As we have outlined in this piece, this involves a number of challenges and opportunities that we will continue to embrace in the future.

## CONCLUSION

Microbiology has long been at the forefront of research and has attained many achievements in diverse areas of life science and medical practice. Its popularity has gained new heights in recent years, not only due to its ever-increasing applicability, but also due to the unprecedented threat of rising antibiotic and antifungal resistance, as well as due to the re-emergence of old infectious diseases and the emergence of new ones. Indeed, microorganisms represent both opportunity and threat. As the Editors of *Microbial Cell*, we have the responsibility to ensure adequate selection of publications that represent a high level of contemporary science. At the same time, we have the ambition to provide an open interdisciplinary communication space for microbiologists from all subdisciplines around the globe, in the interest of scientific and societal progress.
